# Recombinant Expression of Archaeal Superoxide Dismutases in Plant Cell Cultures: A Sustainable Solution with Potential Application in the Food Industry

**DOI:** 10.3390/antiox11091731

**Published:** 2022-08-31

**Authors:** Marta Gogliettino, Stefania Arciello, Fabrizio Cillo, Anna Vittoria Carluccio, Gianna Palmieri, Fabio Apone, Rosa Luisa Ambrosio, Aniello Anastasio, Lorena Gratino, Antonietta Carola, Ennio Cocca

**Affiliations:** 1 Institute of Biosciences and BioResources, National Research Council, Via Pietro Castellino 111, 80131 Napoli, Italy; 2 Arterra Bioscience SpA, Via B. Brin 69, 80142 Napoli, Italy; 3 Institute for Sustainable Plant Protection, National Research Council, Via G. Amendola 122/D, 70126 Bari, Italy; 4 Novamont SpA, loc. La Fagianeria snc, Piana di Monte Verna, 81100 Caserta, Italy; 5 Department of Veterinary Medicine and Animal Production, University of Naples Federico II, 80137 Napoli, Italy

**Keywords:** antioxidant protection, simulated gastric fluid resistance, proteolytic resistance, hyper-thermophiles, tomato cell cultures, food preservation

## Abstract

Superoxide dismutase (SOD) is a fundamental antioxidant enzyme that neutralises superoxide ions, one of the main reactive oxygen species (ROS). Extremophile organisms possess enzymes that offer high stability and catalytic performances under a wide range of conditions, thus representing an exceptional source of biocatalysts useful for industrial processes. In this study, SODs from the thermo-halophilic *Aeropyrum pernix* (SOD*_Ap_*) and the thermo-acidophilic *Saccharolobus solfataricus* (SOD*_Ss_*) were heterologously expressed in transgenic tomato cell cultures. Cell extracts enriched with SOD*_Ap_* and SOD*_Ss_* showed a remarkable resistance to salt and low pHs, respectively, together with optimal activity at high temperatures. Moreover, the treatment of tuna fillets with SOD*_Ap_*-extracts induced an extension of the shelf-life of this product without resorting to the use of illicit substances. The results suggested that the recombinant plant extracts enriched with the extremozymes could find potential applications as dietary supplements in the nutrition sector or as additives in the food preservation area, representing a more natural and appealing alternative to chemical preservatives for the market.

## 1. Introduction

The ubiquitous enzyme superoxide dismutase (SOD) transforms superoxide radicals (O_2_^−.^) into H_2_O_2_, which is successively converted into H_2_O and O_2_ by the enzyme catalase. Unlike the antioxidant chemical scavengers, SOD enzymes are characterized by intrinsic renewal capacities, which means that they do not get exhausted but can keep their catalytic activity for prolonged periods of time [1]. SODs are classified in Fe-SOD (found in prokaryotes and in some plant chloroplasts), Mn-SOD (in prokaryotes and in mitochondria), and Cu/Zn-SOD (in all eukaryotes, including animals and plants), according to the type of metal ion associated to the catalytic site [2]. Thanks to their versatile properties, these enzymes have been proposed for a wide range of industrial applications. However, like most enzymes, their use in food is limited as they require specific ranges of chemical and physical conditions to work properly and effectively [3,4]. Most of the SODs belonging to mesophile organisms often have low stability and easily lose their antioxidant capacity when exposed to the environment [5]; thus, it has been proposed that the protection of the conformational structure and longer storage stability by either site-directed mutagenesis or by mixing with compatible additives would make them potentially useful for industrial applications [6,7]. In this context, extremophile microorganisms, which thrive in extreme habitats that encompass both physical and geochemical environments, represent an interesting source of stable, highly-active, and resistant SOD enzymes, able to work in harsh conditions such as high and low temperatures or pHs, elevated UV doses, and high levels of salt [6,8,9,10,11,12]. Among these microorganisms, the archaeons *Aeropyrum pernix* and *Saccharolobus solfataricus* have attracted much attention for their unusual habitats and extraordinary resistance properties, as the first can be found in harsh marine environments, such as hydrothermal vents on the deep seafloors [13,14,15,16], and the second in hot volcanic sulfuric ponds [17]. Both archaeons are hyper-thermophilic, proliferating at temperatures ranging from 70 to 100 °C, and in addition, *A. pernix* stands up to 7% salinity [16], while *S. solfataricus* optimally grows at pH from 2 to 4 [18]. The employment of extremophilic antioxidant enzymes in the food sectors, where the presence of salts may affect enzymatic activity, can offer a significant advantage over the use of mesophilic enzymes that are less stable under extreme conditions. Salty food, such as marine fish, can be seriously damaged by oxidation which compromises its organoleptic properties, leading to a rapid deterioration of its appearance, taste, and nutritional value. On the other hand, ingested SOD enzymes, contained in functional foods or supplements, barely resist the human stomach environment, being rapidly inactivated by low pHs and digestive proteases, and thus unable to fulfil their antioxidant action any longer. A solution to these applications can come from the use of extremophilic SODs, like those derived from microorganisms which are naturally adapted to live either in salty or acid environments.

In this study, we cloned and expressed the genes *sod_Ss_* and *sod_Ap_* (homologous to both *sodA* and *sodB* genes from *Escherichia coli*, coding for the manganese- and iron-containing enzymes Mn-SOD and Fe-SOD) from *S. solfataricus* and *A. pernix*, respectively, in tomato (*Solanum lycopersicum*) cells in order to produce plant extracts enriched with SOD*_Ss_* or SOD*_Ap_* and investigate on their structural and biochemical properties. Indeed, a competitive and sustainable solution for the expression of heterologous proteins was represented by the plant cell cultures, as the derived extracts, besides the recombinant proteins, were safe and contained secondary metabolites that might have beneficial effects in a wide range of industrial applications, specifically in food safety and human health.

The transgenic cell extracts exhibited extremophilic properties, resulting in exceptional resistance to salt for SOD*_Ap_* and low pHs for SOD*_Ss_*, together with optimal activity at high temperatures for both enzymes. These findings make them promising natural ingredients to protect against the deleterious effects of oxidation in food preservation and human nutrition. Moreover, we tested the SOD*_Ap_*-expressing tomato cell extracts by evaluating their influence on the fillet colour of thawed yellowfin tuna (*Thunnus albacares*) and fresh bluefin tuna (*Thunnus thynnus*), monitoring the histamine contents at different times. The trial on processed tuna fish can be considered a pilot study elucidating the potential advantages of using natural antioxidants in a complex system like defrosted tuna over commercial brine solutions.

## 2. Materials and Methods

### 2.1. Cloning of Aeropyrum Pernix K1 and Saccharolobus Solfataricus P2 Mn/Fe-SOD Coding DNA Sequences (CDS)

Genomic DNAs from *Aeropyrum pernix* K1 (taxonomy ID: 272557) and *Saccharolobus solfataricus* P2 (taxonomy ID: 273057) were isolated from frozen cells using the PureLink^®^ Genomic DNA Mini Kit (Invitrogen/Thermo Fisher Scientific, Waltham, MA, USA). The CDS of the two *sod* genes (locus *sodF*_AERPE and *sodF*_SACS2, respectively), encoding the Mn/Fe-dependent SOD (UniProtKB accessions Q9Y8H8 and P80857, respectively), were amplified by PCR. The amplification mix was as follows: 25 ng of genomic DNA as a template, 0.02 U/μL of Phusion Green HotStart II High-Fidelity DNA Polymerase (Thermo Fisher Scientific, Waltham, MA, USA), 0.5 μM of each primer (Table S1A), and 200 μM of each dNTP. The PCR reactions were performed following the conditions already reported in Palmieri et al. [11]. The generated amplicons were confirmed by sequencing at Eurofins Genomics (Ebersberg, Germany). The 645 bp *sod_Ap_* and 636 bp *sod_Ss_* products were subcloned into the blunt end pSC-B-amp/kan vector using the StrataClone PCR Cloning Kit (Agilent Technologies, Santa Clara, CA, USA) and the sequence verification of the final constructs was carried out (Figure S1A,B).

### 2.2. Cloning of Sod_Ap_ and Sod_Ss_ in Plant Expression Vectors

Both *sod* coding sequences were cloned into pCK-EGFP [19] by replacing EGFP with the *sod* sequences. The constructs were modified by the addition of four restriction sites (KpnI, SalI, SpeI, SmaI) at the 3′ of the 35S terminator (terK3S). The *sod_Ap_* Open Reading Frame (ORF) was amplified by PCR from the pSCB-*sod_Ap_* cloning vector using the primers *sod_Ap_*_NcoI_F and *sod_Ap_*_BX_R (Table S1B) and the purified product was cloned into the NcoI-XbaI sites of pCK-EGFP. The *sod_Ss_* ORF was amplified by PCR from the pSCB-*sod_Ss_* vector with primers *sod_Ss_*_NcoI_F and *sod_Ss_*_BX_R (Table S1B), and the purified product was cloned into the NcoI-XbaI sites of pCK-EGFP. The expression boxes of two pCK vectors included a tandem duplication of the 250 bp upstream sequences of the TATA elements of the CaMV 35S promoter which acted as a strong enhancer of protein expression. For in planta expression of *sod_Ap_* and *sod_Ss_*, the binary plasmid pBI121 was modified by replacing the existing GUS expression cassette with those inserted in the previously described pCK-*_Ap_sod*terK3S and pCK-*_Ss_sod*terK3S. The expression cassette for *sod_Ap_* and *sod_Ss_* under the control of the CaMV 35S promoter and terminator sequences (between HindIII and SmaI restriction sites) were briefly subcloned into pBI121 digested with HindIII and EcoRI (Figure S2). The accuracy of the subcloning procedures was controlled by nucleotide sequencing on the newly synthesized vectors.

### 2.3. Genetic Transformation of Tomato Plants by Agrobacterium Tumefaciens

The *sod* genes, subcloned into the expression vector pBI121, were transferred to the plant cells via *Agrobacterium tumefaciens* transformation, according to the procedure described previously [11]. The primers used to amplify the *sod_Ss_* or *sod_Ap_* are reported in Table S1C.

### 2.4. Molecular Analysis of the Tomato Transformed Lines

The presence of the two transgenes in the tomato transformed lines was verified following the same protocol reported in Palmieri et al. [11]. Western Blot (WB) analysis was carried out on the total proteins which were extracted from 100 mg of tomato calli ground in the extraction buffer consisting in 150 mM Tris-HCl, pH 7.4, 150 mM NaCl, 1.5 mM EDTA, 1.5% Triton X-100, and a mixture of protease inhibitors with a broad specificity for the inhibition of serine, cysteine, aspartic proteases, metalloproteases, and aminopeptidase (MERCK, Darmstadt, Germany). Hence, the homogenates were centrifugated at 15,000× *g* for 15 min at 4 °C to remove cellular debris, and the supernatants were used as crude cell extracts. The total protein content was determined by using the Bio-Rad protein assay kit (Bio-Rad, Hercules, CA, USA). The immuno-blot was performed as described below.

### 2.5. Production of Tomato Cell Extracts

Plant cell cultures were initiated using the protocol already described in Palmieri et al. [11]. The lyophilised powder was dissolved in water at a concentration of 10% *w*/*v*, and the protein concentration was measured by the Bradford assay [20], using bovine serum albumin (BSA) as standard.

### 2.6. SOD and NBT Assays

SOD activity was measured spectrophotometrically based on the inhibition of the Nitro Blue Tetrazolium (NBT) reduction [21]. Briefly, the reaction mixture (1 mL), containing 2 µg of the enzyme extracts obtained from non-transformed (WT, wild-type) or sod-transformed tomato cells, Tris-HCl 50 mM, pH 8.0, 0.1 M EDTA and 1.5 mM NBT was exposed to a light source for 8 min. Following the exposition, 0.12 mM riboflavin was added, and the solution was exposed to the light for an additional 12 min to initiate the photochemical reaction. The reaction was stopped by switching off the light source, and absorbance was measured at 560 nm using a Jasco 640-V spectrophotometer equipped with a temperature control unit. One unit of SOD activity was defined as the amount of enzyme required to produce a 50% inhibition of NBT reduction under assay conditions. Protein concentration was estimated by the Bradford method [20], using BSA as the standard. The effect of pH on SOD*_Ss_* activity was examined by incubating protein samples (2 µg) in 50 mM buffers at different pH values (1.0–8.0) for 15 min at 37 °C: pH 1.0–3.0, glycine-HCl; pH 5.0, sodium acetate; pH 8.0, Tris-HCl. The effect of ionic strength on the SOD*_Ap_* activity was evaluated by incubating protein samples in 50 mM Tris-HCl, pH 8.0 for 15 min at 37 °C at different NaCl concentrations (0.5–1.5 M). The effect of temperature was measured by performing the SOD activity assay after incubation for 15 min at temperatures ranging from 60 to 90 °C. The SOD activity in all the experimental conditions was assayed as described above. Fold increase was calculated as a ratio between SOD activity in crude protein extracts of wild-type plants and SOD activity in crude protein extracts of transgenic plants, using the NBT assay. The relative activity was expressed as a percentage of the corresponding maximal activities under the standard assay conditions. All experiments were performed in triplicate on three different protein preparations.

An in-gel SOD assay was performed as described in Beauchamp and Fridovich [21], with slight modifications. Briefly, an equal amount of each sample was separated by electrophoresis on 10% non-denaturing polyacrylamide gel (Native-PAGE). Then, the gel was soaked in a 50 mM Tris-HCl buffer (pH 8.0) containing 0.305 mM of NBT and 0.275 mM of riboflavin for 15 min in the dark at room temperature, followed by incubation in 50 mM Tris-HCl buffer, pH 8.0, and 0.1% TEMED for an additional 15 min under the same conditions. The SOD bands were visualized as a clear white region in the purple formazan background after illumination with a fluorescent lamp.

### 2.7. Molecular Mass Determination

The size exclusion chromatography, performed on a Superdex 200 column (Pharmacia Biotech, Milan, Italy), was used to estimate the native molecular mass of the partially purified SODs. The column was pre-equilibrated with 50 mM Tris−HCl buffer (pH 7.5) containing 50 mM NaCl. The SOD proteins were partially purified from the transformed tomato cell extracts by two subsequent steps of ultrafiltration using molecular weight cut-off (MWCO) spin filters of 100-kDa and 30-kDa (Millipore, Burlington, MA, USA). The retentates were loaded on the column. Standard protein markers (BioRad code 151–1901) were used to calibrate the gel filtration column.

### 2.8. In Vitro Gastric Digestion Assay on SOD_Ss_-Enriched Tomato Cell Extracts

The in vitro gastric digestion assay was conducted as reported in Corcoran et al. [22], with modifications. Briefly, simulated gastric fluid (SGF) was formulated using NaCl (2.05 g L^−1^), KH_2_PO_4_ (0.60 g L^−1^), CaCl_2_ (0.11 g L^−1^), and KCl (0.37 g L^−1^), adjusted to pH 2.0 using HCl and autoclaved at 121 °C for 15 min. Porcine pepsin (13.3 mg L^−1^) was added as a stock solution prior to analysis. For all assays, freshly prepared, simulated digestion fluid was used. In the applied gastric digestion procedure, 30 µg of protein extracts derived from both non-transformed (WT, wild-type) and *sod*-transformed tomato cells were mixed in a ratio of 1:4 (*v*/*v*) with SGF without or with pepsin and incubated at 37 °C for 3 h. The pH was re-adjusted with 1 M HCl during digestion. 30 µg of WT extract in buffer at pH 7.5 was used as control. After incubation, the reaction mixtures (40 µL) were analysed by NBT-PAGE. The same digestion procedure was followed using BSA (30 µg) as protein control. In this case, the reaction mixtures were analysed by SDS-PAGE (12%). Standard proteins (broad range) were from New England BioLabs (Ipswich, MA, USA).

### 2.9. Resistance of SOD_Ss_-Enriched Tomato Cell Extracts to Proteolytic Degradation

The proteolytic stability of SOD*_Ss_* was assessed by incubating 30 µg of protein extracts derived from both non-transformed (WT) and *sod*-transformed tomato cells with Trypsin (10 µg and 20 µg) or Chymotrypsin (10 µg and 20 µg) at 37 °C for 3 h. Then, the reaction mixtures (40 µL) were analysed by NBT-PAGE. The same procedure was followed using BSA (30 µg) as protein control, and the reaction mixtures were analysed by SDS-PAGE (12%).

### 2.10. SDS-PAGE and Western Blot Analyses

Thirty micrograms of the total proteins were loaded into 12% SDS-PAGE gel, and the immunoblotting was performed on polyvinylidene fluoride (PVDF) membrane (Millipore) by using the specific anti-SOD antibody, diluted 1:10,000 in TTBS buffer (Tris-buffered saline and 0.05% tween 20) containing 5% Blotting-Grade Blocker (Bio-Rad, Hercules, CA, USA). A goat anti-rabbit antibody conjugated to horseradish peroxidase (HRP) was used as a secondary antibody (dilution 1:5000), and the membrane was developed by a Pierce TM 1-Step Ultra TMB blotting solution (Thermo-fisher, Waltham, MA, USA). The commercially available MnSOD*_Ec_* from *E. coli* (SRP6107 MERCK, KGaA, Germany) was used as a control.

### 2.11. ORAC Assay

The spectrophotometric quantification of the inhibition of myoglobin (Mb) peroxidation was used to assess the antioxidant capacity of the tomato extract enriched in SOD*_Ap_* by a high-throughput 96-well microplate assay. Specifically, the chemical damage to Mb by peroxyl radicals generated by the thermal decomposition at 37 °C and pH 7.4 of the azo initiator AAPH was measured as a decrease in its intrinsic absorption at 409 nm. The method was conducted in 50 mM phosphate buffer (pH 7.4) at 37 °C, where 100 µL of Trolox standard solutions (5–100 mM) or plant cell extracts (WT and SOD*_Ap_*) and 100 µL of myoglobin (75 mg/mL) were mixed in each well. Then, the microplate was pre-incubated at 37 °C for 30 min. 100 µL of freshly prepared AAPH solution (180 mM) was added, and the absorbance at 409 nm was recorded every minute for 40 min. The antioxidant activity of the extracts was determined according to the method already described by Huang et al. [23]. Oxygen radical absorbance capacity (ORAC) values of the samples were expressed as micromoles of Trolox equivalents per gram.

### 2.12. Sampling Preparation

Chilled yellowfin tuna (*Thunnus albacares*) and fresh bluefin tuna (*Thunnus thynnus*) fillets were bought at local markets in Southern Italy. Bluefin tuna was filleted in the laboratory and each fillet was cut into portions of the same size and weight (4516 ± 10.5 g). Experimental brines were inoculated by using a multi-needle industrial brine injector (Metalbud Nowicki MHM-39/156) (Figure S3) and manually set as follows: brine pressure needle at 1.3 atm, the head speed at 30 cycles/minute, and conveyor belt speed at 75 mm/cycle. The fillets, placed on a conveyor belt, were injected with different brines, while control samples (CTR) were injected with an aqueous solution. Once weight stabilisation was achieved, each sample was packed under a vacuum and stored at refrigeration temperature (2 ± 1 °C) for 10 days. Several samples were created: yellowfin tuna fillets treated with the commercial brine solution (CM), a vegetable extract prepared by dissolving 7.5 g in 1 L of water, as recommended by the manufacturer; yellowfin and bluefin tuna fillets treated with SOD*_Ap_* and WT solutions. All analyses were carried out at days 0, 6 and 10.

### 2.13. Colour Measurements

The changes in the colour of tuna fillets over time were followed by using the Konica Minolta CM-2500d colourimeter (Minolta Co., Ltd., Osaka, Japan) with observer 10° (CIE64) and illuminant D65 as the main measuring conditions set. The colour measurements were conducted, including a specular component (sci mode) and adopting the CIELAB colour space: lightness (L*), redness (a*), and yellowness (b*). Total colour difference (Δ*E*) and variation in a* (Δa*) were calculated to better describe the colour changes that occur during the storage period as follows:

(1) $$\Delta E=\;\sqrt{{\left(L^{*}{}_{1}-L^{*}{}_{2}\right)}^{2}+{\left(a^{*}{}_{1}-a^{*}{}_{2}\right)}^{2}+{\left(b^{*}{}_{1}-b^{*}{}_{2}\right)}^{2}}$$

(2) $$\Delta a*=a^{*}{}_{2}-a^{*}{}_{1}$$

where L*_2_, a*_2_, and b*_2_ were the values recorded on a specific day during the storage; instead, L*_1_, a*_1_, and b*_1_ were the values collected at day 0. For the colorimetric study of the fresh bluefin and thawed yellowfin tuna over the storage time, the evaluation of the inner surface was carried out after 10 min of its exposure to air. To obtain representative results, four superficial measurements were performed on the surface of tuna slices because the colour might not be homogeneous over the entire surface. Specifically, imagining the major surface of a tuna slice as a triangular figure, the colour measurements were performed on areas corresponding to the three angles and the centre.

### 2.14. Histamine and Nitrate Determination in Tuna Fillets

The histamine content was measured using a commercial enzyme immunoassay test kit (RIDASCREEN^®^ Histamine enzymatic, R-Biopharm, Darmstadt, Germany) according to the manufacturer’s instructions. The histamine concentration (μg/kg) was calculated with the RIDASOFT^®^ Win.NET software. The measurement of the nitrate levels in yellowfin tuna fillets was conducted according to the method reported by Cortesi et al. [24]. On each sample, the analyses were performed in triplicate the day after the injection of brines.

### 2.15. Statistical Analysis

Experiments were carried out in triplicate, and results were expressed as means ± standard error. Data were statistically analysed with a generalised linear mixed model (GLMM) through SPSS version 27 (IBM Analytics, Armonk, NY, USA) with brine type and storage times as a fixed effect. Tukey’s HSD post-hoc test was used to calculate the significant differences between means at a significance level of *p* < 0.05.

## 3. Results and Discussion

### 3.1. Expression of SOD_Ap_ and SOD_Ss_ in Tomato Cell Cultures

**Figure 1:**
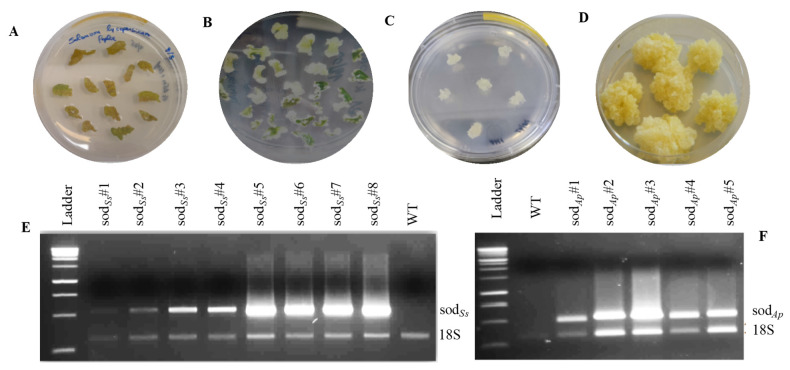
Transgenic calli production from tomato cotyledons (cv MicroTom) and RT-PCR analysis of recombinant SODs. (**A**) *Callus* induction in cotyledon pieces; (**B**) *callus* formation on MS medium containing NAA and kinetin; (**C**) *callus* lines regenerated; (**D)** transgenic clones; RT-PCR analysis of (**E**) *sod*_*Ss*_ and (**F**) *sod_Ap_* transgenic lines. Ladder: 1 Kb DNA marker (Promega, Madison, WI, USA); WT: untransformed tomato calluses; 18S: amplification product of internal standard 18S gene.

Firstly, the genes coding SOD*_Ss_* and SOD*_Ap_* were cloned following the methods described in the Materials and Methods section and sequenced to confirm their identity. The coding sequences were expressed under the control of a variant of the 35S promoter, which included a TEV leader (TL) sequence and duplication of 250 base pairs upstream of the 35S TATA element [19], to induce a ten-fold increase in the transcriptional activity and an enhancement of the translation efficiency respect to that of the natural promoter [25]. Moreover, the incorporation of additional unique sites at the end of the terminator facilitated the sub-cloning of the full expression cassettes into the binary expression of plasmid pBI121. Cotyledonary leaves of tomato cv MicroTom were co-cultivated with *Agrobacterium tumefaciens* cultures carrying the binary vectors pBICK-*sod*_*Ss*_ and pBICK-*sod_Ap_* for plant transformation (Figure S2). After about four weeks, calli, originating from selective media from the explants, were transferred to specific media to allow their growth and maintain the callus state (Figure 1A–D). Hence, semi-quantitative RT-PCR was performed to detect the expression of the *sod* genes in the different transgenic lines. The presence of an amplification band corresponding to transgenic *sod_Ss_* (Figure 1E) or *sod_Ap_* (Figure 1F) genes was evidenced in all the analysed cell lines, differently from the untransformed tomato cells (WT).

**Figure 2:**
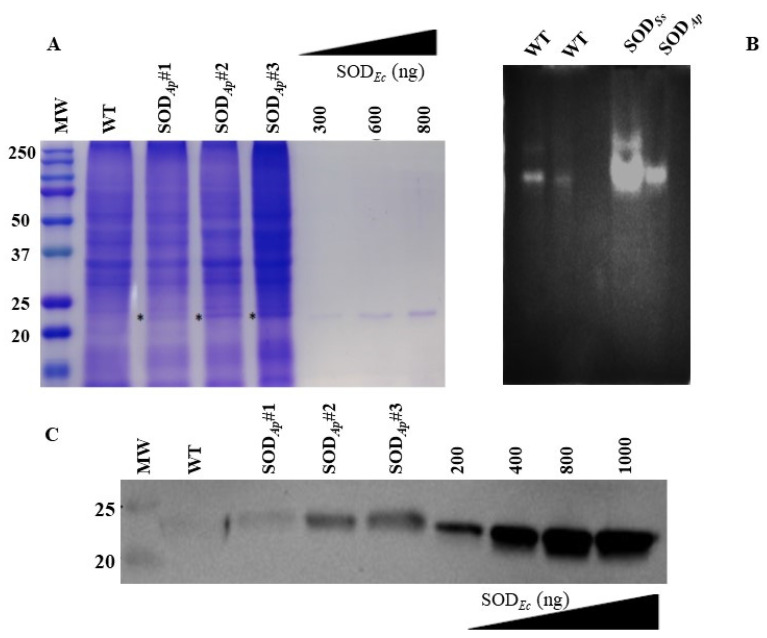
SDS-PAGE, NBT-PAGE and Western blot analyses of total protein extracts from transgenic tomato cell lines. (**A**) Total protein extracts (30 µg) were electrophoresed on 12% SDS-polyacrylamide gel and detected with Coomassie blue staining. (**B**) Total protein extracts from tomato cell lines untransformed (WT) (first lane: 15 μg and second lane: 7 μg) or transformed with *sod_Ss_* and *sod_Ap_* genes (15 μg) were electrophoresed on Native-PAGE (10%). Following Native-PAGE, protein bands were detected by in-gel SOD activity staining using the Riboflavin-NBT assay. The results are representative of three independent experiments on three different protein extracts. (**C**) Total proteins (15 μg) were extracted from the tomato cell lines, separated by SDS-PAGE and transferred to PVDF membrane. WB analysis was carried out using anti-SOD antibody. WT: untransformed tomato cells; SOD*_Ap_*#1, SOD*_Ap_*#2 and SOD*_Ap_*#3: callus lines transformed with *sod_Ap_* gene. SOD*_Ec_*: commercially available SOD from *E. coli* was used for the comparative quantification of the relative intensity of band of interest indicated with * in (**A**).

The transformed callus lines were then subcultured, and total proteins were extracted. Then, the crude extracts were analysed by SDS-PAGE, NBT-PAGE, and Western blotting to confirm the presence of the expressed proteins. As shown in the SDS-PAGE reported in Figure 2A, an extra protein band with the expected molecular mass of 24.5 kDa was visualised in all the analysed SOD*_Ap_* transgenic samples compared to the protein profile of the wild-type. This band was also positive for Native-PAGE followed by in-gel SOD activity (Figure 2B) and Western blot analysis by using the specific anti-SOD antibody (Figure 2C), thus confirming the presence and the nature of the recombinant protein in the plant transgenic extracts. To roughly estimate the percentage of transgenic SOD*_Ap_* in the total soluble proteins (TSP) of tomato cells, iBrigtTM software was used for the densitometric analysis and SOD*_Ap_* was quantified by using the commercially available SOD from *Escherichia coli* at known concentrations as reference (Figure 2A). It was not possible to perform the same analysis by Western blot as exogenous and endogenous SOD isoforms were undistinguishable by the anti-SOD Ab. The percentage of SOD*_Ap_* accumulated in transgenic tomato cells was estimated at around 1.2–1.5% (*w*/*w*), in agreement with data reported in the literature [26]. The *sod_Ss_* product was expressed at undetectable levels in all the differently obtained transgenic cell lines from SOD*_Ap_*. Nevertheless, the *S. solfataricus* enzyme was detected in the transgenic plant extracts by Native-PAGE followed by in-gel SOD activity in the transgenic plant extracts, thus confirming the presence of the recombinant protein (Figure 2B). This technique has the advantage of being extremely sensitive as levels of 10–100 ng of enzyme can already be detected.

Such a result could not be surprising considering that the genes from the archaeon *S. solfataricus* contain high proportions of rare codons, which often reduce the levels of their heterologous expression [27], and the detectability of the gene products by Western blot analysis is under the detection limit. To overcome this limitation, future investigations could focus on an alternative strategy based on synthetic gene design with optimised codon usage for plants to increase the amount of enzyme production in the extracts.

Finally, the molecular mass of both partially purified SODs was determined under native conditions by gel-filtration chromatography, using a Superdex 200 column calibrated with proteins of known molecular size. As shown in Figure S4A, SOD*_Ss_* eluted as a single symmetric peak with an apparent Mr of ~52 kDa, according to the calibration curve and to the electrophoretic analyses followed by enzyme activity staining (Figure S4B). This value approached the theoretical mass of a homodimer, thus confirming that the extremophilic enzyme had a structural organisation which consisted of two identical subunits of approximately 24 kDa when expressed in tomato cells. The same result in terms of the oligomeric organization was obtained with the partially purified SOD*_Ap_* (data not shown).

### 3.2. Biochemical Characterization of SOD_Ap_ and SOD_Ss_

**Figure 3:**
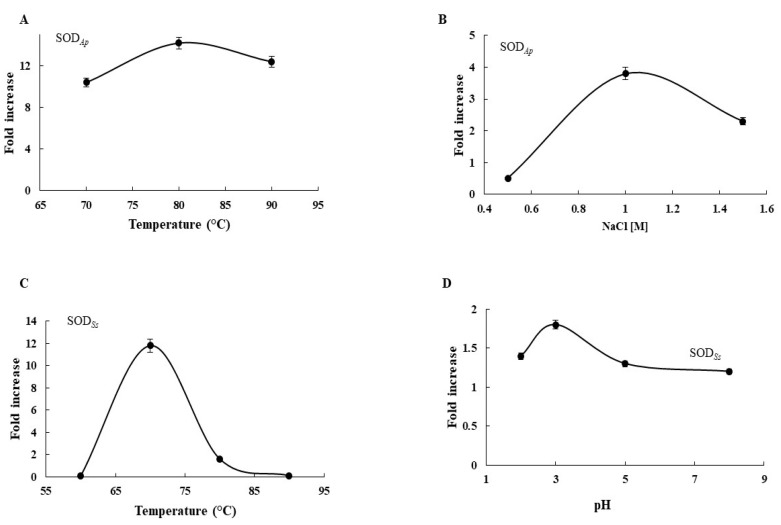
Biochemical analysis of transgenic extracts. Effects of (**A**) temperature and (**B**) NaCl on SOD*_Ap_* activity. Effects of (**C**) temperature and (**D**) pH on SOD*_Ss_* activity. Fold increase was calculated as ratio between SOD activity in crude protein extracts from wild-type plants and SOD activity in crude protein extracts from transgenic plants, using the NBT assay. All experiments were performed in triplicate on three different protein preparations. Data were expressed as means ± standard deviation. Standard deviation values lower than 5% were not shown.

To evaluate the potential applications of the two extremozymes in industry, the effects of diverse physicochemical effectors such as temperature, pH, and saline concentration on SOD activity were examined in the transgenic protein extracts using the Riboflavin-NBT assay. As depicted in Figure 3, SOD activity in SOD*_Ap_*-transgenic extracts was 10.4- to 12.4-fold greater than that in the WT in the temperature range from 70 to 90 °C (Figure 3A), with the maximum registered at 80 °C. Moreover, the enzymatic activity in the transgenic protein samples was 0.5- to 2.3-fold higher than that in WT under the tested NaCl concentrations (Figure 3B). Similar results were also obtained when protein extracts from SOD*_Ss_*-tomato lines were incubated at high temperatures or different pHs. Indeed, the SOD activity increased under the investigated conditions in recombinant extracts with respect to that in the untransformed cell lines, with the major effects observed at 70 °C (11.8-fold) (Figure 3C) and pH 3.0 (1.8-fold) (Figure 3D). These results suggested that the expression of the extremophilic SODs conferred to the transgenic cell extracts the resistance to extreme conditions compared to non-transgenic ones, in accordance with the hypertermo-halophilic and hypertermo-acidofilic nature of SOD*_Ap_* and SOD*_Ss_* isoforms, respectively.

**Figure 4:**
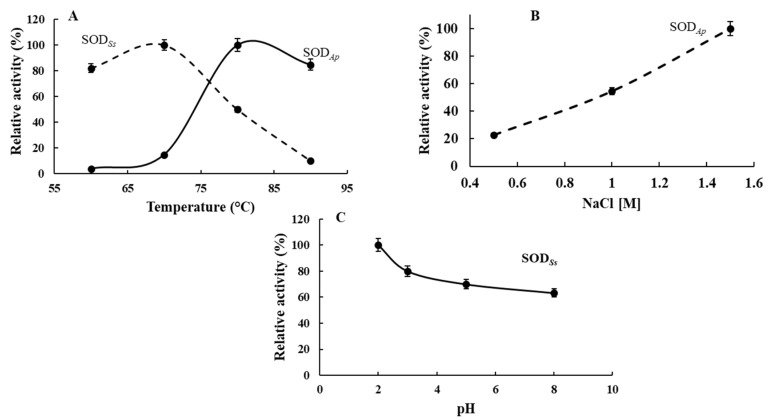
Biochemical properties of SOD*_Ss_* and SOD*_Ap_*. Effect of (**A**) temperature on SOD*_Ap_* and SOD*_Ss_* activity and (**B**) NaCl on SOD*_Ap_* activity. Effect of (**C**) pH on SOD*_Ss_* activity. The activity at the optimal temperature, pH or NaCl concentration was defined as 100%. The SOD activity was measured using the NBT assay, and all experiments were performed in triplicate on three different protein preparations. Data were expressed as means ± standard deviation. Standard deviation values lower than 5% were not shown.

Furthermore, the characterisation of the biochemical properties of the recombinant SODs carried out under the specific environmental conditions in which their organisms thrive revealed that SOD*_Ss_* and SOD*_Ap_* showed their optimum activity at 70 and 80 °C, respectively, in agreement with their hyperthermophilic origin (Figure 4A). However, SOD*_Ss_* and SOD*_Ap_* still kept more than 80% of their activity at 60 and 90 °C, respectively, with a linear reduction observed between 80 and 90 °C for SOD*_Ss_* and 60 and 70 °C for SOD*_Ap_*. Concerning SOD*_Ap_*, the influence of saline concentration was also investigated due to its marine source. As reported in Figure 4B, SOD*_Ap_* activity concomitantly increased with salinity, reaching its optimum at 1.5 M NaCl concentration. Finally, SOD*_Ss_* characterisation was performed in a pH range from 2.0 to 8.0, showing a slow decrease of its activity as the pH value increased, with the maximum measured at pH 2.0, thus reflecting the acidophilic nature of the extremophilic enzyme (Figure 4C).

### 3.3. Resistance of SOD_Ss_-Tomato Cell Extracts to Simulated Gastric Fluid (SGF) and Pancreatic Proteases

Since most proteins are susceptible to digestive proteolysis (pepsin, pancreatic endoproteases), which compromises their structural integrity and function, there is an urgent need to find good solutions to protect therapeutic enzymes from gastric acid and proteolytic digestion. Indeed, it has been reported that SODs-enriched plant extracts developed as a dietary supplement often fail because the low pH and the high proteolytic activity in the digestive tract could be chemically inactive and thus render the enzyme ineffective [28]. Therefore, the use of extremozymes with exceptional stability features could represent an interesting alternative to circumvent this problem.

**Figure 5:**
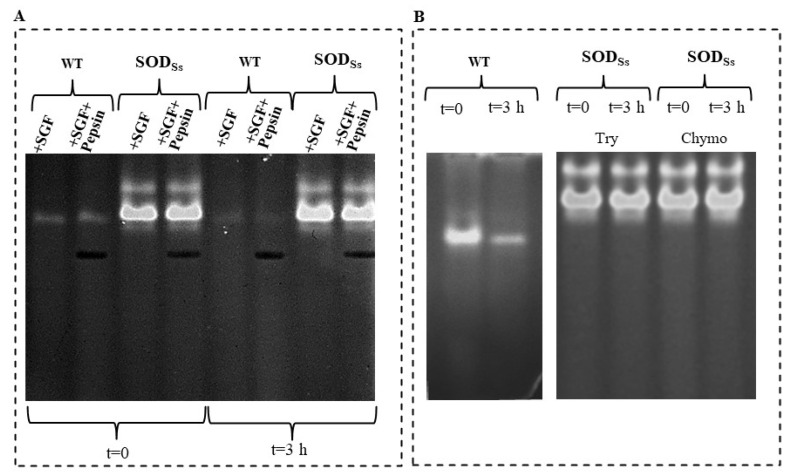
Proteolytic resistance of SOD*_Ss_*-enriched plant extracts. (**A**) Total protein extracts (30 µg) obtained from non-transformed tomato cell lines (WT) or transformed with *sod_Ss_* (SOD*_Ss_*) non-incubated (*t* = 0) or incubated for 3 h at 37 °C with simulated gastric fluid (SGF) in the presence or absence of pepsin (9 µg). (**B**) Total protein extracts (30 µg) obtained from non-transformed tomato cell lines (WT) non-incubated (*t* = 0) or incubated with trypsin (20 µg) or chymotrypsin (20 µg) for 3 h at 37 °C; total protein extracts (30 µg) obtained from tomato cell lines transformed with sod*_Ss_* (SOD*_Ss_*) non-incubated (*t* = 0) or incubated with trypsin (Try, 20 µg) or chymotrypsin (Chymo, 20 µg) for 3 h at 37 °C. Following Native-PAGE (10%) analysis, protein bands were detected by in-gel SOD activity staining using the Riboflavin-NBT method. The results are representative of three independent experiments on three different protein preparations.

In this context, a study was conducted to assess the resistance of the recombinant hyperthermo-acidophilic SOD*_Ss_* to digestion by pepsin in a medium mimicking the gastric milieu in vitro and to proteolytic degradation by pancreatic proteases. To this aim, the SOD*_Ss_*-tomato cell extracts were incubated with a simulated gastric fluid (SGF) for 3 h at 37 °C, in the presence and absence of pepsin, and then analysed by a NBT in-gel SOD assay. As shown in Figure 5A, different from the wild-type protein extracts (WT), which showed a complete suppression of SOD activity after the treatment, the extracts containing the recombinant SOD*_Ss_* preserved their activity, both after treatment with SGF only and in the presence of pepsin, suggesting a high resistance to acid pHs and proteolysis, according to the extremophilic nature of the enzyme. Similar results were obtained when the resistance to pancreatic serine proteases, trypsin, or chymotrypsin was evaluated (Figure 5B). Specifically, when the WT extracts were incubated with each mammalian protease for 3 h at 37 °C, a marked intensity reduction of the SOD activity band was observed, while the SOD activity was completely preserved in the transgenic SOD*_Ss_* extracts in both the conditions investigated, thus confirming the resistance of this enzyme to protease degradation.

As an additional control, the protein bovine serum albumin (BSA), used as a model of a mesophilic protein, was completely digested when incubated with SGF and the proteolytic enzymes, confirming the susceptibility to degradation by proteases of mesophilic proteins in the tested condition (Figure S5).

### 3.4. Activity of SOD_Ap_-Tomato Cell Extracts on Tuna Slices

The consumption of tuna fish is widespread all over the world, although consumers’ trust is sometimes challenged by the illegal use of additives or unknown substances capable of masking the state of freshness of tuna and preserving its red colour [29]. Indeed, the bright red colour of tuna is considered the main sensory parameter that determines its acceptability in the market and guides the consumers’ choices, acquiring the role of the most economically important factor. Therefore, stakeholders have been researching and using substances able to stabilize the colour of tuna meat for several years now, including banned molecules, such as carbon monoxide, which could mask the degradation progress of tuna and thus its histamine content [30,31].

To test the preservation capacity of fish meat, the extract obtained from the tomato cell line transformed with *sod_Ap_* was tested on fillets of thawed yellowfin tuna (*Thunnus albacares*) and fresh bluefin tuna (*Thunnus thynnus*), by evaluating its influence on the fish colour and monitoring the histamine content during the storage period.

**Figure 6:**
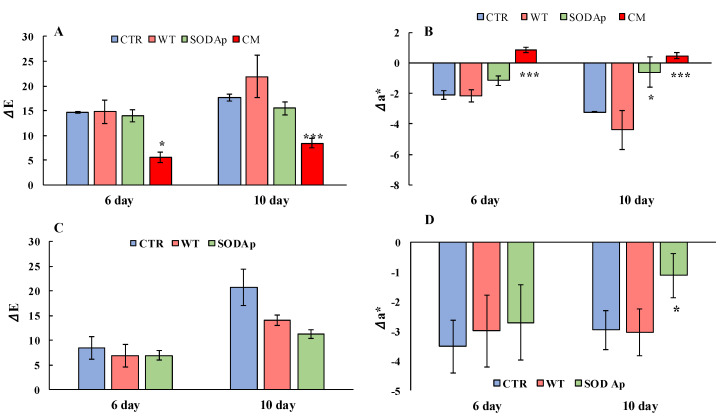
Analyses of total colour difference (Δ*E*) and variation in a* (Δa*) in *Thunnus albacares* and *Thunnus thynnus* fillets during the storage (4 ± 1 °C) for 10 days. (**A**) Δ*E* and (**B**) Δa* values in *Thunnus albacares* fillets injected with aqueous solutions containing water only (CTR: blue); extracts from tomato cell lines non-transformed with *sod_Ap_* (WT: light red); extracts from tomato cell lines transformed with *sod_Ap_* (SOD*_Ap_*: green); commercial brine (CM: red). (**C**) ΔE and (**D**) Δa* values of *Thunnus thynnus* fillets injected with aqueous solutions containing water only (CTR: blue); extracts from tomato cell lines non-transformed with *sod_Ap_* (WT: light red); extracts from tomato cell lines transformed with *sod_Ap_* (SOD*_Ap_*: green). The measurements were conducted on the inner surface of the tuna fillets after 10 min exposure to air. Results are means of three independent experiments, and error bars represent the standard error (sem). * Significant difference (*p* < 0.05) between the treated and the control samples. *** Significant difference (*p* < 0.001) between the treated and the control samples.

Regarding the analyses on the thawed yellowfin tuna fillets, an increasing value of the total colour difference (Δ*E*) over time was detected, because of the colour deterioration that normally occurs. Indeed, the Δ*E* value was strictly connected to the changes in the pigmentations of tissue and, consequently, to all the coordinates of CIElab colour. Specifically, the highest values of Δ*E* were reached by the WT samples (extracts from non-transformed cell lines) followed by the control ones (CTR, injection of only water). The CM (commercial brine) and SOD*_Ap_*-tuna samples exhibited the lowest grade of browning (Figure 6A), confirming the differences in the general colour appearance of the brines employed in this study. Nevertheless, a more specific parameter for describing the changes in acceptability of tuna fillets is the redness value, which is closely related to the content and status of oxidation of myoglobin and haemoglobin [32]. As reported in Figure 6B, the values of Δa* (variations in redness) suggested an important activity of the commercial brine to significantly enhance the red colour of fillets. This result highlighted its alarming ability to mask the natural degradation of the tuna, which retained its freshness properties even when the legislative limit for histamine could be exceeded. Indeed, although the sensory properties were preserved only in CM samples on the twelfth day of storage (data not shown), the analysis of histamine showed higher concentrations (124.2 ± 2.6 mg/kg) compared to the minimum limit set at 100 mg/kg by the Reg. (CE) no. 2073/2005. This commercial powder, which was probably a plant extract of beet, was recognised for its naturally high nitrate content, which could act in the food matrix as a chemical additive. Concerning the level of nitrate detected in the samples under study, none of them exceeded the concentration of 7 mg/kg, with the CM samples showing the highest concentration (5.33 ± 1.16 mg/kg), which was very close to the average amount of nitrate detectable in fish (5 mg/kg) [29,33].

In this regard, it was interesting to note that the use of a low concentration of this commercial powder was dangerous not for its nitrate content but for its ability to stabilise the colour over time, adulterating the tuna and hiding its real state of conservation. Hence, the tomato cell lines transformed with *sod_Ap_* (SOD*_Ap_*) can be considered a promising tool that could guarantee the preservation of the red colour without representing a problem for public human health (Figure 6B). Indeed, our results demonstrated that the efficiency of SOD*_Ap_* brine in improving the tuna colour up to the 10th day of storage, when the histamine values were found below the legislative limits, was due to the presence of the extremophilic SOD enzyme, as the WT samples had no effect (Figure 6).

**Figure 7:**
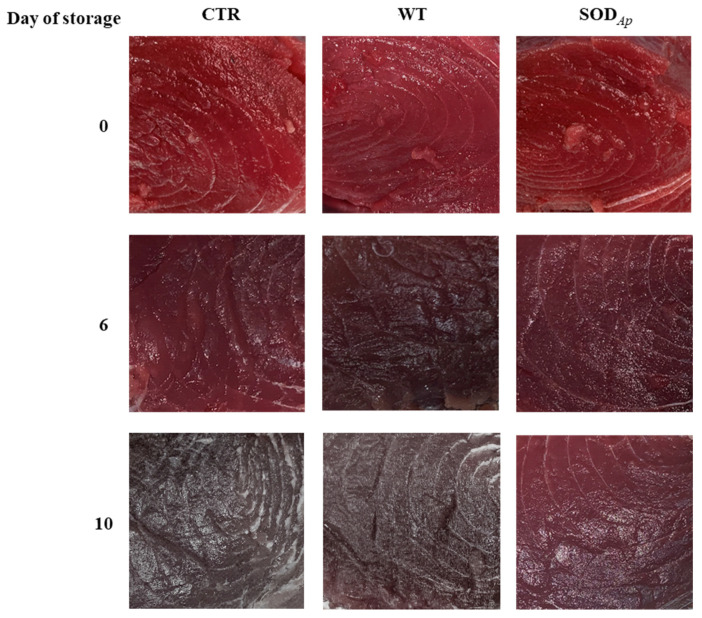
Appearance of *Thunnus thynnus* slices stored for 10 days at refrigerated temperature (4 ± 1 °C). The samples were injected with aqueous solutions containing water only (CTR), extracts from tomato cell lines non-transformed with *sod_Ap_* (WT), extracts from tomato cell lines transformed with *sod_Ap_* (SOD*_Ap_*).

Another set of experiments was conducted to investigate the antioxidant potential of SOD*_Ap_* extracts in bluefin tuna (*T. thynnus*), which was richer in slow fibres and myoglobin compared with *T. albacares* [34]. To this aim, WT and SOD*_Ap_* extracts were injected into fresh bluefin tuna fillets using the water only as control (CTR), and the colour evolution was followed up to the 10th day of storage. As reported in Figure 7, although slight colour differences among samples were already detectable after 6 days of storage at refrigeration temperature, a notable effect of SOD*_Ap_* brines was observed on the fillets after 10 days. Specifically, the values of colour differences (Δ*E*) and the redness variations (Δa*) clarified the potential effects of SOD*_Ap_* brine in slowing down the deterioration processes, which affected the general appearance of fresh tuna fillets (Figure 6C). Considering the colour evolution (Δ*E*) that considered the initial state of tuna fillets pigmentation, significant differences between the colour of each sample were found. Indeed, the Δ*E* values in samples injected with the SOD*_Ap_* brine were lower than those recorded for the other samples (Figure 6C). However, although a weak effect of the tomato extract alone on the product’s general appearance was observed, the values of Δa* referred to WT samples made evident that the decreased Δ*E* did not contribute to the conservation of red colour. On the contrary, significant differences (*p* < 0.05) were found comparing the Δa* values of SOD*_Ap_*-tuna samples with those of control and WT-treated fillets over 10 days of storage (Figure 6D).

In conclusion, the injection of SOD extremozymes in fresh and thawed tuna fillets could guarantee an extension of the products’ shelf life without resorting to illicit substances. To date, the only additives allowed for this type of product have been the antioxidants (ascorbic acid, sodium ascorbate, calcium ascorbate), whose use has been limited to 300 mg/kg by the current European Commission rules.

### 3.5. Activity of SOD_Ap_-Tomato Cell Extracts on Myoglobin Oxidation

As the loss of the red colour in fish meat is mainly linked to the oxidation of oxymyoglobin (OxyMb), an ORAC assay was performed to assess the ability of SOD*_Ap_*-containing extract to protect myoglobin from oxidation, using the compound 2,2′-Azobis(2-amidinopropane) dihydrochloride (AAPH) as oxidant [35]. Scalar dilutions of WT and transgenic SOD*_Ap_* tomato extracts were incubated with myoglobin in the presence of AAPH, and the absorbance decay, due to the oxidation of the heme group of the protein caused by the oxidant, was measured at 409 nm. The values of antioxidant capacity, expressed as micromole of Trolox equivalents per gram, were calculated as explained in the Materials and Methods section and resulted in 388 ± 24 µmol Trolox eq·g^−1^ for the SOD*_Ap_* extract and 195 ± 21 µmol Trolox eq·g^−1^ for the WT extract, indicating that the presence of the enzyme in the tomato cell extract significantly increased the myoglobin protection against oxidation and confirming the results on the redness preservation of fish slices showed above.

## 4. Conclusions

After microbial spoilage, the oxidation of food, resulting in the loss of organoleptic properties and nutritional values, is the second most important cause of food impairment [36]. For this reason, antioxidants have become very popular and scientifically interesting compounds due to their benefits in many areas.

Specifically, in nutrition technology, the enrichment of various foodstuffs with synthetic compounds such as t-Butyl-4-HydroxyAnisole (BHA) or other typology of antioxidants (e.g., vitamins, carotenoids, polyphenols) is a frequently adopted method to counteract the oxidation phenomena. However, many of these additives cannot be used in large quantities as they can be health hazards and cause serious side effects. While vitamins are not stable over time and are subject to hydrolytic processes mostly determined by high temperatures or high salt concentrations [37]. Therefore, one of the main challenges in the food industry is how to minimise the impact of processing techniques and storage methods on food quality due to the increase in consumers’ perception and awareness. In this scenario, extremophilic microorganisms represented an underutilised and innovative source of novel enzymes, having developed unique mechanisms to survive under a wide range of extreme and inhospitable environmental conditions in terms of temperature, pH, or salinity, among others. The extremophile-derived enzymes, or extremozymes, offer new alternatives for biotechnological applications [38], able to perform reactions under harsh conditions, like those found in several industrial processes.

In this paper, protein extracts enriched with SODs from two extremophiles and heterologously expressed in tomato cell lines were biochemically characterised. This innovative approach, which combines the advantage of using plant cells as a bio-factory of recombinant proteins with the extraordinary catalytic properties of extremozymes, could support the development of sustainable, eco-friendly, and efficient food technologies with positive impacts on human health.

## Data Availability

The data presented in the study are available in this manuscript.

## Supplementary Materials

The following supporting information can be downloaded at: https://www.mdpi.com/article/10.3390/antiox11091731/s1. Table S1: Primer sequences used in this study; Figure S1: Coding DNA sequences (CDS) of two extremozymes inserted in the subcloning pSCB vector; Figure S2: Schematic representation of pBICK-*sod_Ss_*/*_Ap_*; Figure S3: Scheme of the experimental procedure for the sampling and preparation of tuna fillets (filleting, brine injection, and vacuum skin packaging-VSP); Figure S4: Gel filtration chromatography of partially purified SOD*_Ss_*; Figure S5: Effect of proteolytic treatments on BSA.
