# Supplementary material for: Recombinant Expression of Archaeal Superoxide Dismutases in Plant Cell Cultures: A Sustainable Solution with Potential Application in the Food Industry
Source: Antioxidants (Basel). 2022 Aug 31;11(9):1731. doi: 10.3390/antiox11091731 (PMC9495943; doi:10.3390/antiox11091731)
Supplement: Supplementary file 1 [file antioxidants-11-01731-s001.zip › antioxidants-1852014-supplementary.pdf]

Table S1. Primer sequences used in this study

A)

N.	Name	Sequence 5'-3'	Ta (°C)	Amplicon size
1	Sod*Ap* for	ATGGTGAGCTTTAAGAGGTAC		
2	Sod*Ap* rev	CTACTGGGGGAGCAGG		
			56	645 bp
3	Sod*Ss* for	ATGACTCTCCAAATTCAGTTTAA		
4	Sod*Ss* rev	TTACTTCGTTAAATATTTCTGTAAC		
			53	636 bp

B)

N.	Name	Sequence 5'-3'	Ta (°C)	Amplicon size
1	EGFPseq-for	GAAGCGCGATCACATGG		
2	35STer-KSSS-rev	<u>CCCGGGACTAGTGTCGACGGTACC</u>TGTCACTGGATTTTGGTTTTAGG		
			52	311 bp
3	SOD*Ss*-NcoI-for	GGG<u>CCATGG</u>CTCTCCAAATTCAGTTT		
4	SOD*Ss*-BX-rev	CCC<u>TCTAGAGGATCC</u>TTACTTCG		
			55	636 bp
5	SOD*Ap*-NcoI-for	GGG<u>CCATGG</u>TGAGCTTTAAGAGGT		
6	SOD*Ap*-BX-rev	CCC<u>TCTAGAGGATCC</u>CTACTGGGGGAGCAG		
			60	645 bp

Restriction sites are underlined.

C)

N.	Name	Sequence 5'-3'	Ta (°C)	Amplicon size
1	SOD*Ap* for	CTTTAAGAGGTACGAGCTCC		
2	SOD*Ap* rev	GTGCTCCCACACGTCAATAAC		
			53	498 bp
3	SOD*Ss* for	GAGCTACCTCCATTACCCTAC		
4	SOD*Ss* rev	CTTCTTTTCCGCTGCATCCC		
			53	581 bp

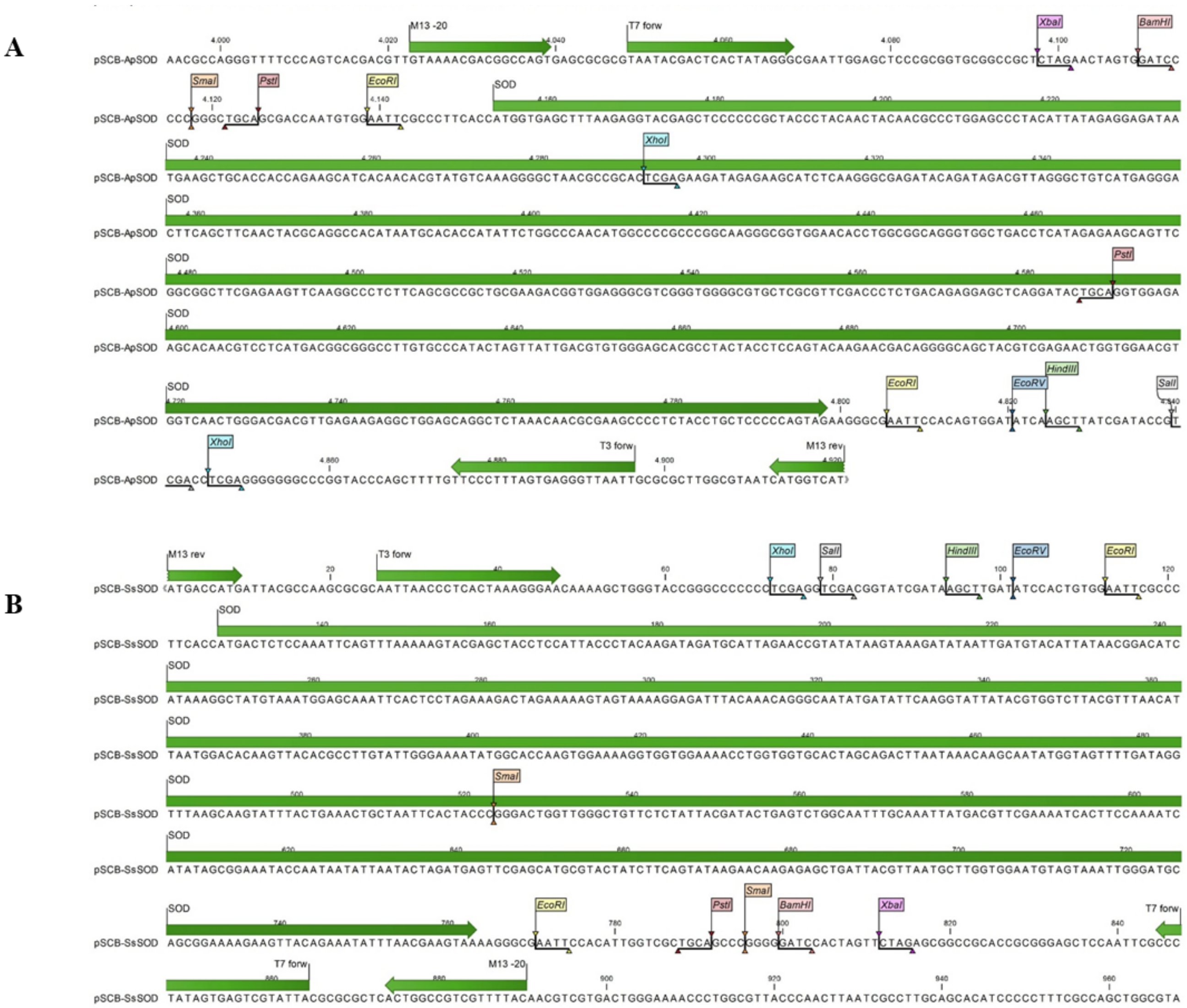

Figure S1. **Coding DNA sequences (CDS) of two extremozymes inserted in the subcloning pSCB vector.** (**A**) SOD$_{Ap}$ and (**B**) SOD$_{Ss}$ CDS

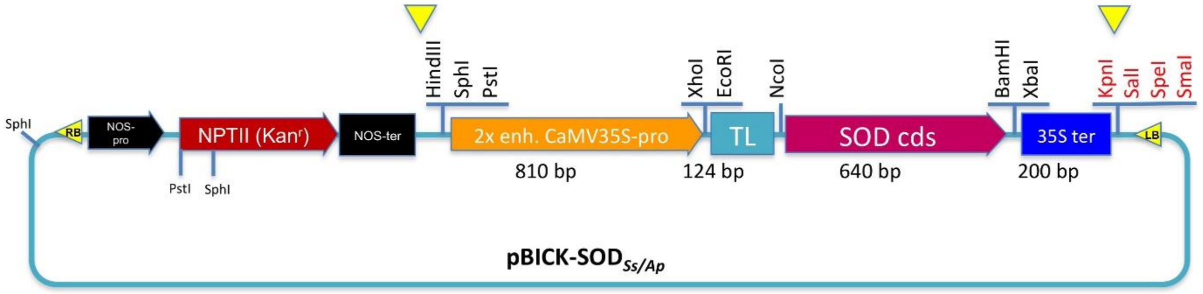

Figure S2. Schematic representation of pBICK-SOD*Ss*/*Ap*. The vector was assembled by introducing between the HindIII/EcoRI restriction sites of the original pBI121 binary vector the expression cassette from pCK expression vector encoding both genes of interest (pCK-SOD*Ap*terK3S and pCK-SOD*Ss*terK3S, described in Materials and Methods) under the control of double-enhancer 35S (2x) promoter and a transcription leader sequence (TL). Transcription stops thanks to the terminator of CaMV 35S.

2x enh. CaMV35S-pro: double-enhancer 35S (2x) promoter from Cauliflower mosaic virus 35S; 35Ster: Cauliflower mosaic virus 35S terminator; SOD*Ap*: *Aeropyrum pernix* SuperOxide Dismutase; SOD*Ss*: *Saccharolobus solfataricus* SuperOxide Dismutase; NPTII (kanr): neomycin phosphotransferase II gene for kanamycin resistance; NOS: nopaline synthase; RB: right border and LF: left border.

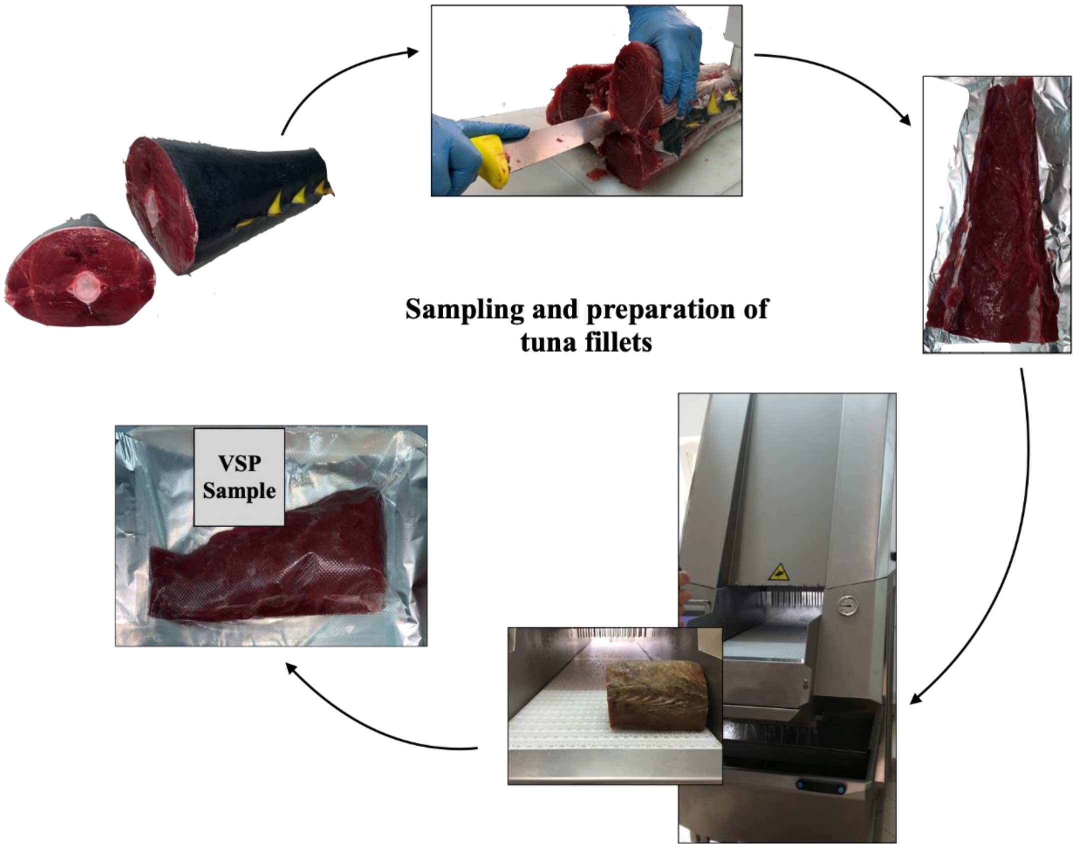

Figure S3. Scheme of the experimental procedure for the sampling and preparation of tuna fillets (filleting, brine injection, and vacuum skin packaging-VSP)

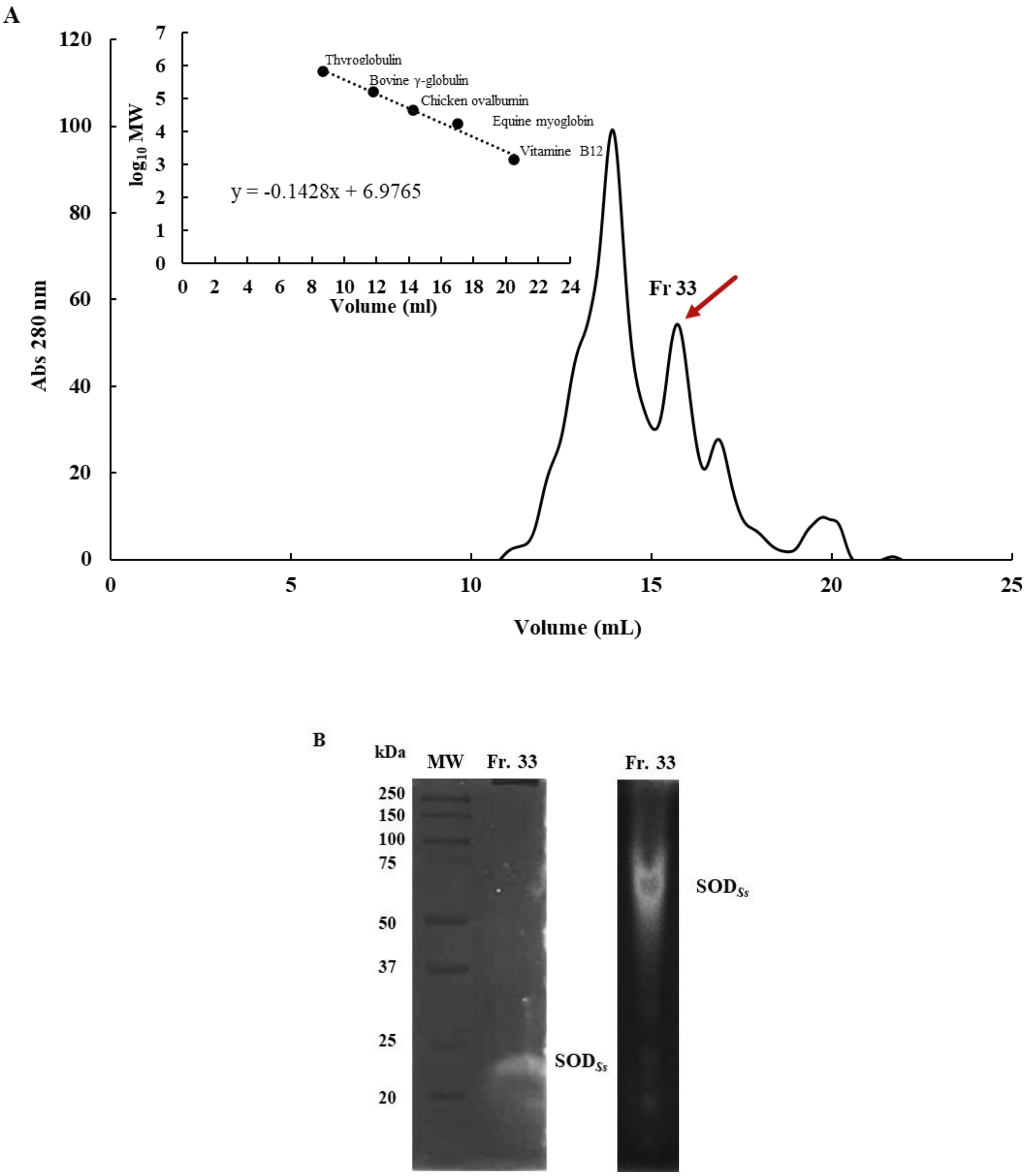

Figure S4. Gel filtration chromatography of partially purified SOD_{Ss}. (**A**) Size-exclusion chromatogram of partially purified SOD_{Ss} on Superdex 200 column in 50 mM Tris-HCl buffer pH 7.5 containing 50 mM NaCl. The absorbance was measured at 280 nm. *Insert*: calibration curve of Superdex 200 column using protein standards of known molecular masses: Thyroglobulin (670 kDa), Bovine g-globulin (158 kDa), Chicken ovalbumin (44 kDa), Equine myoglobin (17 kDa), Vitamine B12 (1.35 kDa). (**B**) SDS-PAGE (10%) (*left*) and Native-PAGE (10%) (*right*) analyses of SOD_{Ss}-fraction obtained after gel filtration chromatography. MW: molecular markers. Following electrophoretic analyses, protein bands were detected by in-gel SOD activity staining using the Riboflavin-NBT method. The results are representative of three independent experiments on three different protein preparations.

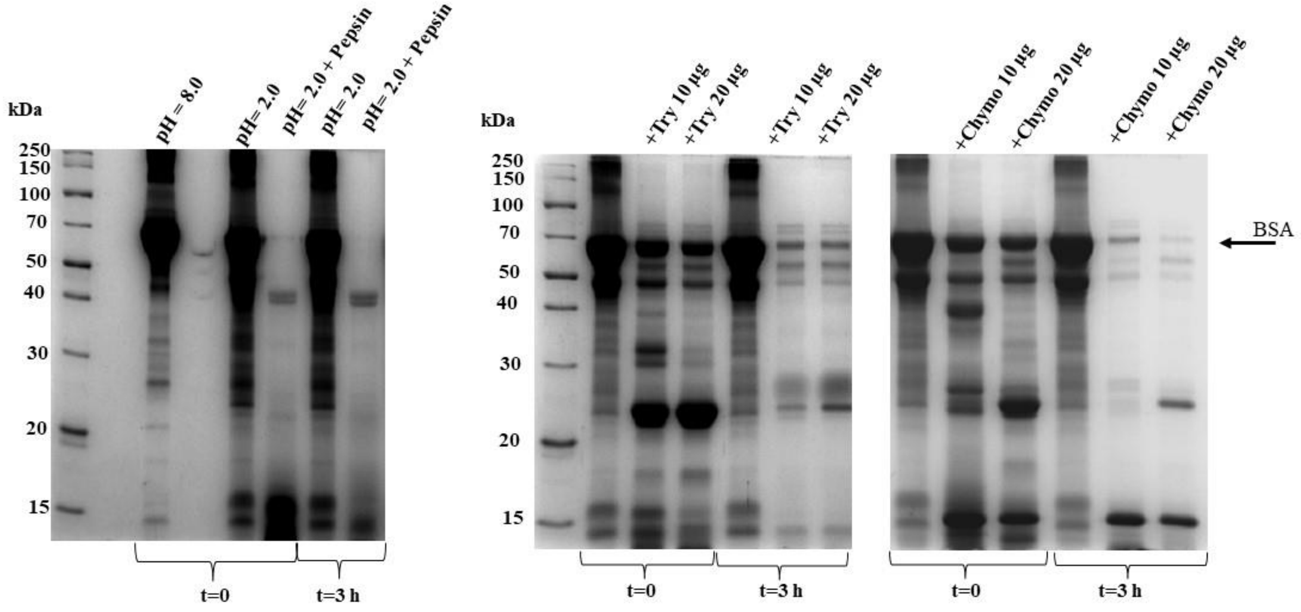

Figure S5. Effect of proteolytic treatments on BSA. SDS-PAGE (12%) analysis of BSA (30 µg) before and after incubation for 3 h at 37 °C with Pepsin (9 µg) at pH 2.0, Trypsin (Try, 10 µg and 20 µg) or Chymotrypsin (Chymo, 10 µg and 20 µg) at pH 8.0. BSA at pH 2.0 and 8.0 was used as control. The results are representative of three independent experiments on three different protein preparations.
